# Cross-Linked Hyaluronic Acid for Cleft Lip and Palate Aesthetic Correction: A Preliminary Report

**DOI:** 10.1093/asjof/ojac052

**Published:** 2022-06-08

**Authors:** Łukasz Ordynowski

**Affiliations:** Dr Ordynowski is an aesthetic practitioner in private practice, Kraków, Poland

## Abstract

**Background:**

Surgical treatment of cleft lip and palate is divided into primary and secondary procedures to restore physiological function and appearance of the face, mouth, and nose. Hyaluronic acid (HA) bio-implants have been successfully used for volume loss correction in several medical disciplines. However, there is paucity of information about its use in the management of facial clefting.

**Objectives:**

The aim of this report is to present the preliminary findings on the feasibility of using a cross-linked HA for aesthetic correction in previously surgical treated cleft lip and palate cases.

**Methods:**

The cross-linked HA STYLAGE L, XL, and XXL (LABORATOIRES VIVACY, Paris, France) were used in this case series. Multiple treatment sessions, 4-6 weeks apart, were performed if required.

**Results:**

A total of 15 patients had undergone the HA injections between May 2018 and December 2021. Of these, 13 had simultaneous correction of the nose, lip, and paranasal scar and the remaining 2 only the lip and scar. The procedures were uneventful and well tolerated by the patients. At follow-up, aesthetic improvement was observed in all patients. Moreover, patients reported overall satisfaction with the outcome of the procedures particularly because of its minimally invasive nature.

**Conclusions:**

Cross-linked HA is a feasible and promising complimentary option for aesthetic, and potentially functional, correction in cases of cleft lip and palate. Larger clinical trials are needed to validate these preliminary findings.

**Level of Evidence: 4:**

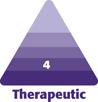

Orofacial cleft anomalies occur secondary to abnormal development of the craniofacial structures and are considered one of the most common birth defects. These anomalies include cleft lip, cleft lip and palate, and cleft palate alone. Although the incidence of these defects varies by gender, race, and socioeconomic conditions, the global prevalence of cleft lip, with or without cleft palate at birth ranges from 3.4 to 22.9 per 10,000 births.^1-3^ Cleft lip and palate tend to be isolated defects in 70% of cases.^4^ Cleft lip occurs due to a developmental disturbance in the formation of the primary palate between weeks 4 and 7 of embryonic life as a result of failure of proper fusion of the maxillary bones with the nasal processes. Although a cleft palate results from disturbance in the formation of the secondary palate between 10 and 12 weeks of pregnancy.^5^ Nevertheless, the underlying etiology of such disturbances is not fully understood. The anatomical disorders caused by the defect(s) can result in multisystem dysfunctions including sucking and swallowing disturbances, nasal obstruction, nasal tone, and articulation distortions and disorders in speech development.^6-9^ Moreover, cleft lip and/or palate has been associated with psychological disorders, low self-esteem, and impaired social interaction. Hence, psychological care for patients and their families should be an integral aspect of a successful management plan.^10^

In general, management consists of a series of surgical operations starting in the first months of a child’s life and continuing well into adulthood. Additionally, patients continue to need multidisciplinary care involving orthodontics, dentistry, mental health, and speech therapy.^11,12^ Surgical treatment of cleft lip and/or palate is divided into primary and secondary reconstructive surgery. The main goal of the primary surgery is to close the cleft fissure to regain the structural basis for sucking, swallowing, chewing, hearing, and speech development and restore the physiological appearance of the face as much as possible. However, secondary operations include restoration of the continuity of the alveolar arch, closure of any oronasal fistulae, palatopharyngeal muscle repair, secondary lip, and nose corrections and orthognathic procedures.^13^ Nevertheless, in view of the invasive nature of surgical reconstruction, it tends to have a significant impact on the individual’s quality of life, additional scarring and is not always associated with favorable outcomes from the patient perspective.^14-16^ Therefore, several studies have explored the possibility of lipofilling, as an alternative aesthetic technique, after facial skeletal maturity has been reached with varying results.^17-21^

Hyaluronic acid (HA) is a polysaccharide from the glycosamino-glycan family and is an extracellular matrix key molecule present in several body tissues. It plays an important role in maintaining tissue integrity and vascularization.^22,23^ HA bio-implants are sterile, biodegradable, viscoelastic, isotonic, transparent injectable gels, which were approved by the Food and Drug Administration (FDA) in 1996 and have since been successfully used for volume loss correction in several medical disciplines. However, there is paucity of information on its use in the management of orofacial cleft anomalies.^24,25^ STYLAGE L, XL, and XXL (LABORATOIRES VIVACY, Paris, France) are nonanimal in origin cross-linked sterile dermal fillers constituting of HA gel together with mannitol with concentrations of 24, 26, and 21 mg/g, respectively. They are designed for use as dermal fillers in cases of deep wrinkles, facial volume restoration, and creation. STYLAGE L, XL, and XXL fillers have been used in our center not only for their classical indications but also for aesthetic correction of previously surgical treated cleft lip and palate cases since 2018. The aim of this report is to present the preliminary findings on the feasibility and patient-reported aesthetic outcomes of using cross-linked HA in this context.

## METHODS

This is a retrospective review of a cohort of patients who were treated by the author between May 2018 and December 2021. Participants included men and women who presented for complimentary aesthetic treatment for a cleft lip with or without cleft palate following prior surgical defect correction(s). This work was undertaken in compliance with the Helsinki declaration. All the patients were provided with detailed information about the procedure and provided a valid written consent. Patients were made aware of the intention to publish this report and have consented to the use of their photographs for this purpose.

The cross-linked HA, STYLAGE L, XL, and XXL were used for the aesthetic procedures. These were administered using either 26 G and 30 G 13-mm needles or 25 G 50-mm and 22 G 70-mm cannulas depending on the area being treated. A cannula was always considered safer to use for nasal reconstructions. When a needle was used, injection was always preceded by aspiration to mitigate the risk of intravascular HA injection. Pre-procedure evaluation included assessing tissue mobility in the area of the bridge and the columella of the nose, the white and red parts of the upper lip, any surgical scars or dental conditions, missing teeth, or occlusal disorders that may affect soft tissue support.

During a treatment session, 2-6 ml of the cross-linked HA was administered. In addition to the lidocaine within some of the used STYLAGE products, analgesia was complemented by a pretreatment application of an anesthetic cream and peritreatment cold compresses. Patients were given information to continue using cold compresses for the first 3 days and to massage the treated area for approximately 4 weeks to facilitate proper spread of the HA and release scarring. Follow-up appointments, either virtual or face-to-face, were organized 4 weeks after treatment to assess the aesthetic outcome and plan additional treatment sessions if required. Once treatment was considered complete, patients’ satisfaction with the global aesthetic outcome, as well as the satisfaction with the correction of the nose, lip, and scar area, was assessed on a 5-point Likert scale where 1 was not satisfied at all and 5 was very satisfied.

## RESULTS

Between May 2018 and December 2021, a total of 15 patients (3 men and 12 women), with an average age of 30.4 years (range 18-52 years) had complimentary reconstruction of their orofacial cleft defects using a cross-linked HA. All the patients had both cleft lip and palate, had previous surgical corrections, and were seen or contacted at their planned follow-up appointments. Patient demographics, number of previous surgical operations, number of HA treatment sessions, volume and type of STYLAGE HA used per session, the facial areas treated with HA, and posttreatment satisfaction scores are presented in Table 1.

**Table 1.** Patient Demographics and Patient-Reported Outcomes of Hyaluronic Acid

Patient ID	Age	No. of previous operations	No. of treatment sessions and the no. and type of STYLAGE (LABORATOIRES VIVACY, Paris, France) used per session	Postoperative satisfaction score		
				Nose	Lip	Scar
1	18	8	1 treatment session; 3 STYLAGE XL	5	5	5
2	36	4	1 treatment session; 3 Stylage XL plus and 3 STYLAGE XL	5	5	5
3	34	4	5 treatment sessions; Session 1: 2 STYLAGE XL, Session 2: 2 STYLAGE XL, Session 3: 1 STYLAGE XXL, Session 4: 1 STYLAGE XL, Session 5: 1 STYLAGE XL	5	5	5
4	51	12	2 treatment sessions; Session 1: 1 STYLAGE XL, Session 2: 1 STYLAGE XL	—^a^	5	5
5	31	11	2 treatment sessions; Session 1: 1 STYLAGE XXL and 1 STYLAGE XL, Session 2: 1 STYLAGE XXL and 1 STYLAGE XL	5	5	4
6	20	4	2 treatment sessions; Session 1: 2 STYLAGE XL, Session 2: 2 STYLAGE XL	5	5	5
7	37	2	2 treatment sessions; Session 1: 1 STYLAGE XL, Session 2: 1 STYLAGE XL	—^a^	5	5
8	27	1	2 treatment sessions; Session 1: 4 STYLAGE XL, Session 2: 2 STYLAGE XL	5	5	5
9	30	14	1 treatment session; 6 STYLAGE XL	5	5	5
10	41	7	2 treatment sessions; Session 1: 3 STYLAGE XL, Session 2: 1 STYLAGE L	5	4	4
11	24	10	1 treatment session; 3 STYLAGE XL and 1 STYLAGE L	5	4	4
12	24	9	1 treatment session; 2 STYLAGE L, 1 STYLAGE M, and 1 STYLAGE XL	5	5	5
13	18	8	1 treatment session; 2 STYLAGE XL	5	5	5
14	40	20	1 treatment session; 7 STYLAGE XXL and 3 STYLAGE XL	4	5	4
15	25	10	2 treatment sessions; Session 1: 1 STYLAGE XXL and 1 STYLAGE XL, Session 2: 1 STYLAGE L	4	5	5

^a^Nose not corrected just lip and paranasal scar.

**Figure 1.**
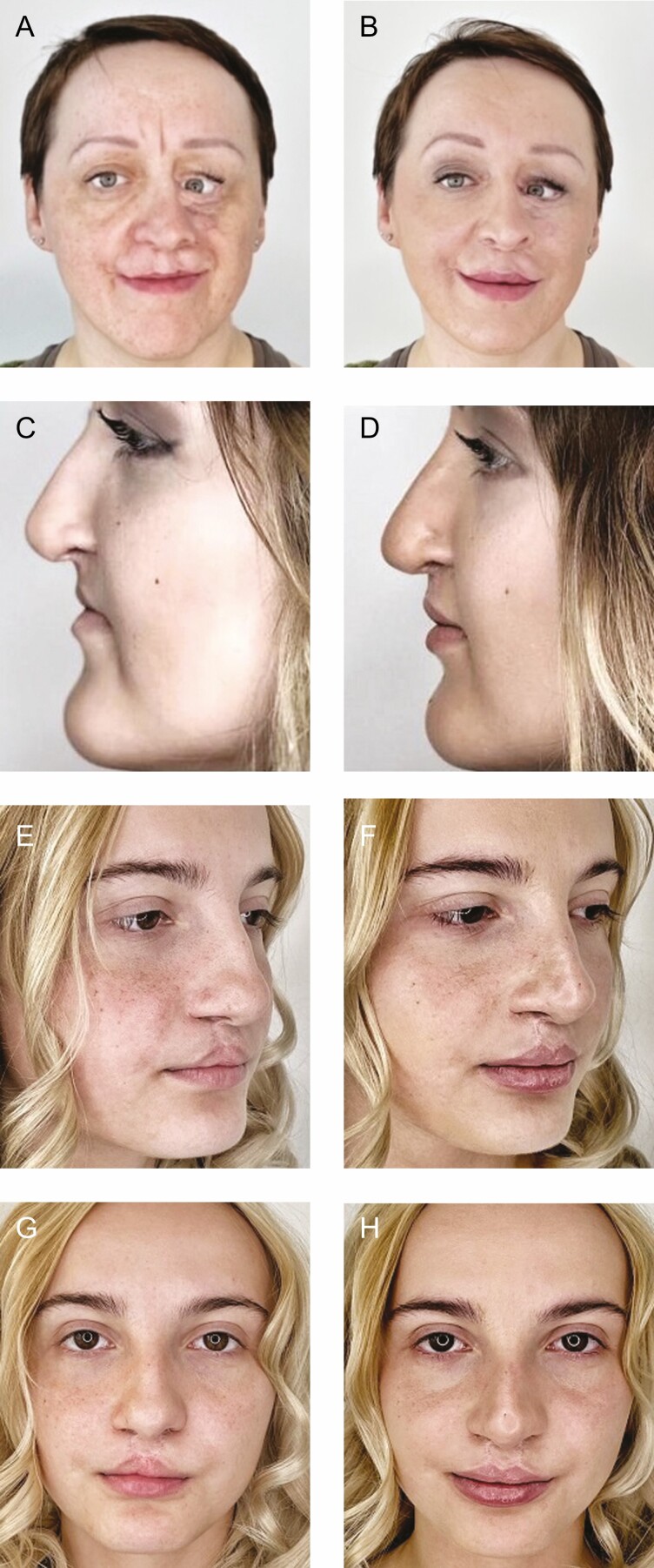
Examples of improvement in lip symmetry after hyaluronic acid injections: 40-year-old female patient shown (A) before the procedure and (B) immediately after the procedure; a 20-year-old female patient shown (C) before the procedure and (D) immediately after the procedure; and an 18-year-old female patient shown at 3/4 view (E) before the procedure and (F) immediately after the procedure, and at frontal view (G) before the procedure and (H) immediately after the procedure.

**Figure 2.**
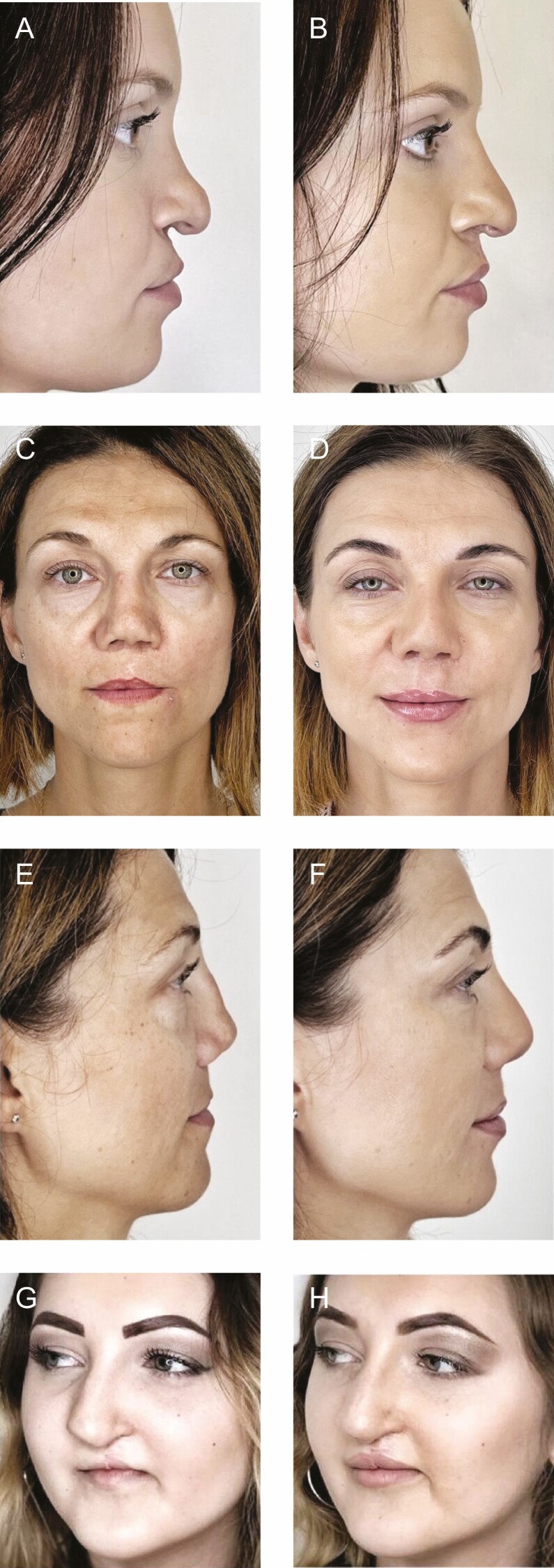
Examples of improvement in the appearance of the nose bridge and columella: a 24-year-old female patient shown (A) before the procedure and (B) immediately after the procedure; a 41-year-old female patient shown at frontal view (C) before the procedure and (D) immediately after the procedure, and at side view (E) before the procedure and (F) immediately after the procedure; and a 20-year-old female patient shown (G) before the procedure and (H) immediately after the procedure.

**Figure 3.**
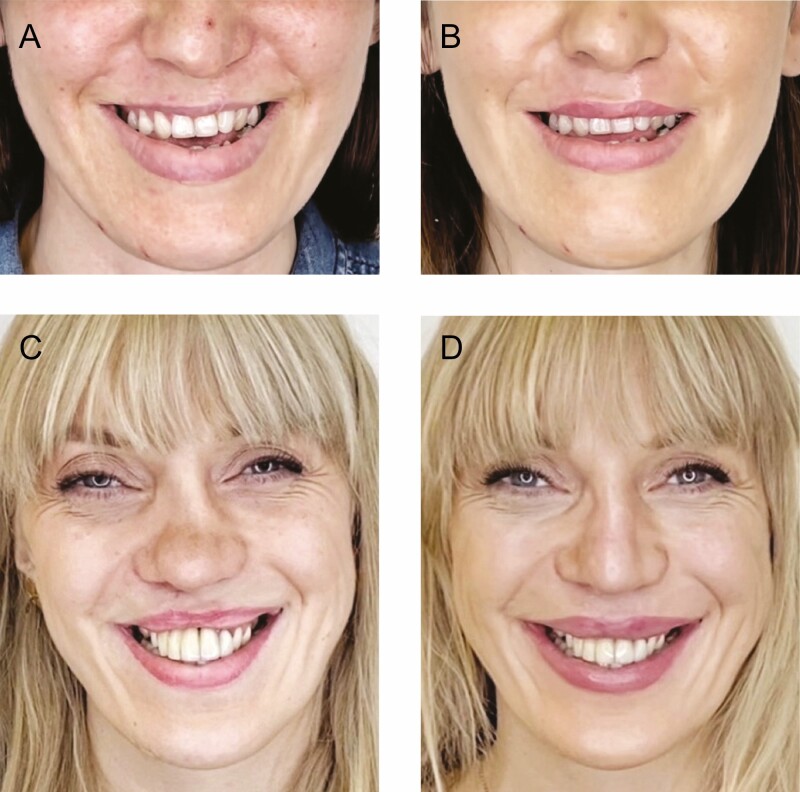
Examples of improved lip closure and smile aesthetics following treatment: a 37-year-old female patient shown (A) before the procedure and (B) immediately after the procedure, and a 30-year-old female patient shown (C) before the procedure and (D) immediately after the procedure.

In general, following the completion of treatment sessions, all the patients reported aesthetic improvement. Overall, the look of the entire face became more harmonious, and the cleft features were reduced in front and side views. On examination, there was partial masking of the bone defect, improvement in lip symmetry (Figure 1), and better appearance of the nose bridge and columella (Figure 2). In some patients, the correction of the red area of the upper and lower lips made it even possible to achieve proper lip closure and improved the smile aesthetics (Figure 3).

Side effects following injections included swelling, bruising, and tenderness. However, these subsided within the first 7 days after the procedure in all patients. No other adverse effects were observed by the treating physician or reported by the patients.

## DISCUSSION

In this preliminary report, the author presents the initial experience with the use of a cross-linked HA dermal filler, developed for use in the management of deep wrinkles and facial volume restoration, as a complementary tool for the aesthetic management of orofacial clefts. In this context, HA is not intended to replace surgical and orthodontic reconstruction but to complement these modalities in improving aesthetic outcomes. In addition to functional outcomes, aesthetic outcomes are an important issue in the management of patients suffering with cleft lip with or without palate because of their impact on the individuals’ mental health, self-esteem, and social interaction.^10^ Indeed, during the follow-up consultation of the cohort of patients included in this report, several expressed their positive views about the impact of the treatment on improving their self-acceptance and how this contributed to a reduction in their perception of the stigma associated with the defects. It is possible that open reconstructive surgery can achieve similar results to what was achieved by the use of HA injections. Nevertheless, the minimally invasive nature of dermal injections has to be factored in when comparing these treatment modalities. Some of the patients in this cohort reflected on their previous surgeries and the associated side effects of such procedures, namely, pain and disruption to their daily normal activities. They even expressed their reluctance to consider surgical procedures given the availability of HA bio-implants as a treatment option.

However, several issues need to be taken into account when assessing such treatment modality. Although the cost of dermal injection in this context will be comparable to its use for other aesthetic procedures, it is important to explore whether such cost might be prohibitive for some self-funding patients especially that multiple treatment and repeat sessions are required. Second, assessing the longevity of treatment effect is important for patients to make an informed choice about their management. Moreover, this information is essential for any accurate health-economic evaluation. A recent experts’ opinion report suggested that the effect of HA in nonsurgical rhinoplasty tends to last 9-12 months if the nose is injected for the first time; however, it lasts up to 3 years if injected for the second time.^26^ One should not extrapolate evidence generated from one procedure to another. Nevertheless, it is plausible that in view of the increased scarring, from multiple previous surgical operations to correct the cleft, the effect of HA fillers in this context might last longer. Third, it is imperative to take into account the safety of the procedure. Filler injections can be associated with several complications ranging from mild bruising and swelling to very serious ones like skin necrosis, particularly when injecting the nose in the presence of excessive scarring.^26,27^ In this study, a cannula was always used for nasal reconstructions, and when a needle was used, injection was always preceded by aspiration to mitigate the risk of intravascular injection.

There are several limitations to this work, which include the small sample size, the fact that it was designed or intended to be a research study to assess the effectiveness of cross-linked HA in the management of orofacial clefts and the lack of use of validated questionnaires to measure clinical outcomes, hence, the preliminary nature of our report. However, this is the largest report on the use of a cross-linked HA bio-implant in this management where previous reports were on isolated case studies.^24,25^ Moreover, it is the first report on the use of STYLAGE L, XL, and XXL in this context.

## CONCLUSIONS

In conclusion, based on these preliminary findings, the use of a cross-linked HA dermal filler (STYLAGE L, XL, and XXL) is a feasible option in the complementary aesthetic management of previously surgically corrected cleft lip and palate. The treatment was well tolerated by patients with no untoward side effects or complications during the follow-up period. Based on the patients’ reported satisfaction with the outcome and its minimally invasive nature, this treatment seems to have a promising potential. However, further larger studies are needed before drawing any final conclusions.
